# Valorization of reactivated carbon and recovered nutrients from gas adsorption filters for sustainable fertilization

**DOI:** 10.1038/s41598-026-64228-x

**Published:** 2026-08-05

**Authors:** Magdy A. Baiomy, Mayada E. Abdel Razek, Osama K. Ahmed

**Affiliations:** 1 Bio-Engineering Dept., Agric. Engineering Research Institute, Agric. Research Center, Dokki, Giza Egypt; 2 Chemical and Biological Technologies in Agriculture, Fac. Agric, Cairo University, Giza, 12613 Egypt

**Keywords:** Spent activated carbon, Nutrient recovery, Gas adsorption filters, Soil fertility, Sustainable agriculture, Waste management, Fertilization, Environmental sustainability, Chemistry, Environmental sciences

## Abstract

In response to growing environmental concerns from industrial and agricultural pollution, sustainable waste management strategies are increasingly important. This study presents a dual evaluation of spent activated carbon (AC) and nutrients recovered from gas adsorption filters, focusing on both fertilizer applications and the valorization of reactivated carbon. Adsorbent materials, such as AC impregnated with magnesium oxides, effectively capture harmful exhaust gases, but their disposal poses environmental challenges. Here, the chemical composition of spent AC was analyzed, and its potential as a soil amendment was assessed. Various nutrient recovery methods, including acidic, alkaline, and water-based extractions, were evaluated for their efficiency in retrieving nutrients from the spent adsorbent. The recovered solutions were chemically characterized based on Fe, K, and Mg concentrations. Results showed that alkaline extraction using 3% KOH yielded the highest in the recovered solution (Fe 7.45%, K 1.89%, Mg 1.22%), highlighting potential for producing nutrient-rich liquid fertilizers. Water-based extraction preserved the AC pore structure most effectively, achieving the highest BET surface area (118.18 m²/g), while acidic HCl treatment was associated with reduced surface area and pore volume, suggesting restricted pore accessibility and possible structural deterioration. Reducing adsorption performance. Germination assays revealed that water-washed filtrates promoted the greatest barley root elongation, whereas KOH filtrates exhibited inhibitory effects, reflecting differences in ionic composition. Economically, water-based regeneration was the most cost-effective (< 10% of virgin AC), while KOH treatment provided the dual benefit of reactivating AC for reuse and producing nutrient-rich solutions. Overall, the findings demonstrate that reused activated carbon coupled with recovered nutrients can enhance sustainable agriculture and support circular waste management systems.

**Supplementary Information:**

The online version contains supplementary material available at 10.1038/s41598-026-64228-x.

## Introduction

This study presents a dual-valorization approach for spent activated carbon (AC) from gas adsorption filters, simultaneously addressing nutrient recovery and carbon reactivation. Unlike previous research that considered these processes separately, this work integrates efficient extraction of recovered nutrients (K, Mg, and Fe) for fertilizer applications with reactivation of AC for reuse in new filtration systems. By combining laboratory analyses of extraction methods, structural characterization, and plant growth assays, the study demonstrates a sustainable, circular strategy that transforms waste into value-added products, providing both environmental and economic benefits while promoting sustainable agriculture and industrial resource reuse.

Industrial and agricultural activities generate a wide range of materials that, after their useful life, eventually become waste that requires effective and sustainable management. Among these, spent adsorbent materials, particularly activated carbon (AC) used in gas purification and water treatment, accumulate adsorbed pollutants and gradually lose adsorption efficiency. Activated carbon is a highly porous carbonaceous adsorbent with a large surface area that enables efficient capture of organic and inorganic pollutants^1^. However, once saturated with contaminants, spent AC becomes a disposal challenge, and traditional methods such as landfilling can pose environmental risks without recovering the material’s inherent value^2^.

In response to these challenges, circular resource management approaches have gained interest. One promising strategy is the recovery of adsorbed nutrients from spent adsorbents, which can provide a sustainable alternative to conventional synthetic fertilizers. Carbonaceous adsorbents, including activated carbon and biochars, have demonstrated strong capacity to capture nutrient ions such as nitrogen, phosphorus, and potassium from waste streams, suggesting potential for nutrient recovery and reuse in agriculture^3^. Extracted nutrient-rich fractions can be processed into fertilizers or soil amendments, enhancing soil fertility and reducing reliance on non‑renewable fertilizer inputs^4^. Broader reviews of circular economy strategies also emphasize the need for reuse and recovery of industrial materials, including both carbon adsorbents and nutrients, as part of sustainable material cycles^5^.

In addition to nutrient recovery, spent AC itself can be reactivated and reused in industrial applications, thereby extending its lifecycle and reducing demand for virgin adsorbent. Regeneration techniques — such as solvent desorption combined with thermal or chemical treatment — have been shown to restore significant adsorption capacity to spent AC, making it suitable for reuse in filtration systems^6^. For example, solvent regeneration and microwave‑assisted reactivation have successfully recovered surface area and pore structure for reuse in water and gas treatment^7^. This dual strategy — valorizing spent activated carbon through regeneration for reuse in filters and extracting nutrients for fertilizer applications — offers a synergistic pathway for reducing waste, conserving raw materials, and supporting sustainable agriculture and industrial practices. This dual strategy — valorizing spent activated carbon through regeneration for reuse in filters and extracting nutrients for fertilizer applications — offers a synergistic pathway for reducing waste, conserving raw materials, and supporting sustainable agriculture and industrial practices. AC regeneration, adsorbents reinforced with magnesium oxide, and nutrient reclamation are all approaches to waste valorization. Researchers have previously studied various approaches to waste valorization, including activated carbon regeneration, adsorbents reinforced with magnesium oxide, and nutrient reclamation.Industrial gas adsorption filters utilizing activated carbon loaded with MgO have received very little attention. Furthermore, few studies have simultaneously evaluated the agricultural potential of recycled washing effluents and the characteristics of regenerated adsorbents^8^. Thus, this research suggests a unified framework for a thorough dual-valorization approach that includes nutrient recovery, regeneration, and evaluation of wasted AC/MgO by means of barley germination assays on the recovered extracts.

Although AC regeneration and nutrient recovery studies are developing, these two techniques have typically been considered different resource recovery routes. Moreover, there is minimal research on the integrated valorization of spent MgO-impregnated activated carbon from industrial gas adsorption filters. In particular, the simultaneous evaluation of nutrient recovery, adsorbent regeneration, and agricultural suitability of the recovered extracts remains underexplored. Thus, a holistic evaluation of regeneration strategies in terms of both nutrient recovery and the recyclability of the adsorbents is still required to facilitate circular economy targets and sustainable waste management. This work studies the potential application of discarded MgO-impregnated activated carbon from gas adsorption filters for dual valorization. In particular, the study assesses the efficacy of different regeneration methods (water, HCl, and KOH) to recover vital nutrients and to restore the physicochemical features of the used adsorbent. Furthermore, the agricultural potential of the recovered extracts was evaluated by germination assays. The regenerated activated carbon was characterized to evaluate the restoration of its physicochemical and textural properties and its potential for reuse in filtration applications.

## Materials and methods

In this study, the potential for reusing spent AC from exhaust gas purification was evaluated using an adsorbent material composed of AC and MgO to purify emissions from the Kubota M100 tractor^9^. To achieve this, three different washing methods—acidic, alkaline, and water washing—were applied to extract nutrients adsorbed onto the carbon surface and produce a nutrient-rich solution for soil enhancement. Additionally, the spent AC was regenerated to restore its adsorption capacity, contributing to the sustainability of gas purification processes. The experimental procedures were systematically conducted through key steps, including the preparation of extraction solutions, heating, filtration and pH adjustment, water washing, and finally, drying and reactivating the activated carbon for reuse in gas purification applications.

The selection of H₂O, HCl, and KOH washing treatments was based on their different regeneration mechanisms. Water washing was selected as a simple and environmentally friendly physical treatment to remove soluble compounds while preserving the carbon structure^10^. HCl treatment was applied to promote the dissolution of inorganic deposits and metal-containing residues accumulated during gas purification^11^. KOH treatment was selected due to its alkaline activation effect, which can enhance pore accessibility and facilitate nutrient recovery from the spent AC/MgO material^12^.

### Extraction methods for adsorbent regeneration

#### Alkaline extraction for nutrient recovery

**Fig. 1:**
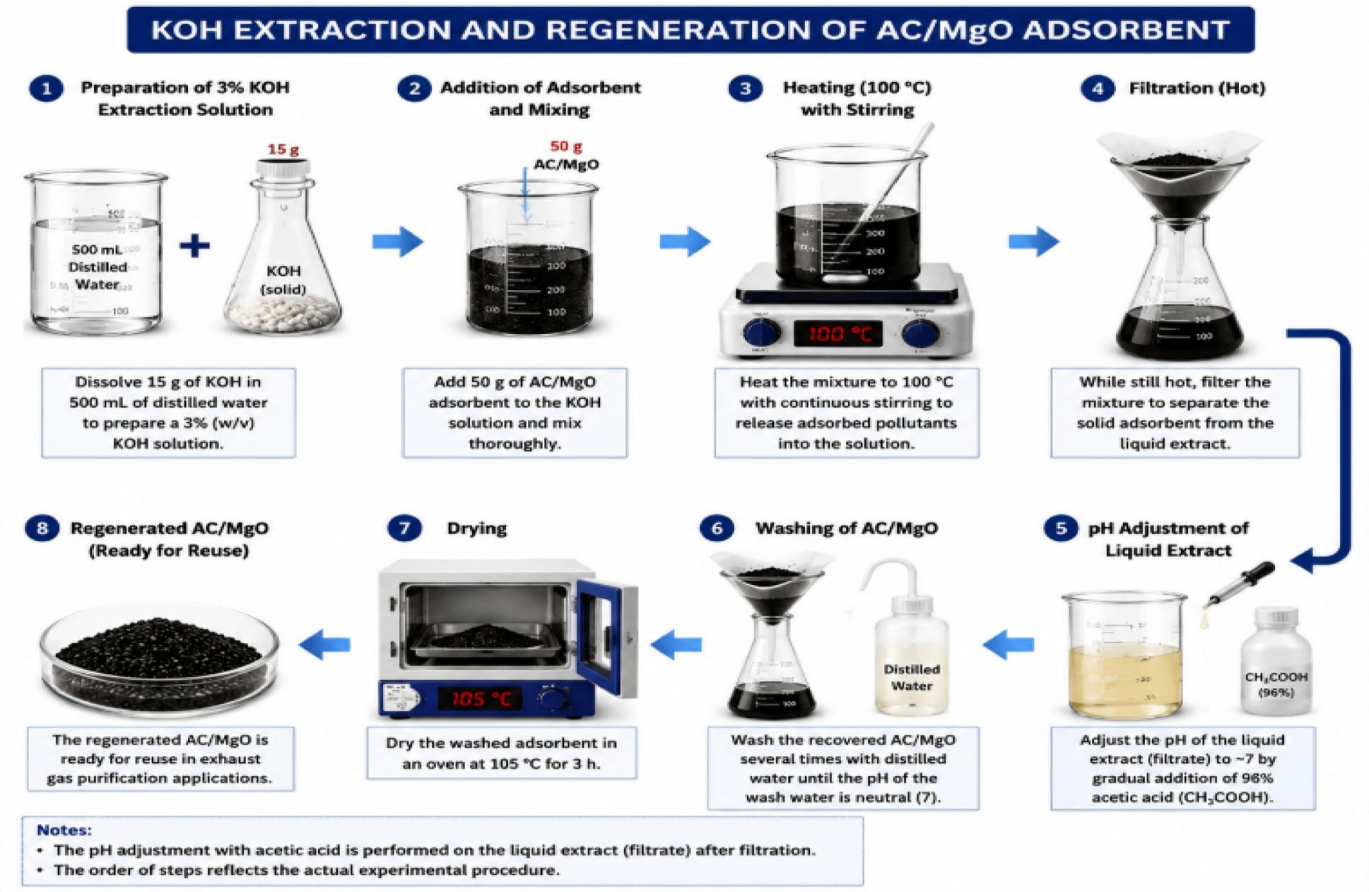
Steps of extraction using KOH and activation of activated carbon: laboratory procedure.

The extraction and analysis of pollutants adsorbed onto the material’s surface involved several key steps. First, a 3% potassium hydroxide (KOH) extraction solution was prepared by dissolving 15 g of KOH in 500 mL of distilled water^13^. Followed by the addition of 50 g of the adsorbent material (AC impregnated with Mg) and thorough mixing. The mixture was then heated to 100 °C using a magnetic stirrer with a heating plate to break the chemical bonds between the gaseous pollutants and the adsorbent surface, facilitating their release into the extraction solution. While still hot, the solution was filtered to separate the solid adsorbent from the extracted pollutants. The pH of the extract was adjusted to approximately 7 using 96% acetic acid (CH₃COOH) to improve solution stability and minimize potential side reactions. prior to subsequent handling and analysis. The AC was then washed multiple times with distilled water until a neutral pH (7) was achieved, ensuring the removal of any residual chemicals. Finally, the adsorbent material was dried in an oven at an appropriate temperature to restore its adsorption capacity, making it suitable for reuse in exhaust gas purification applications. Figure 1. Illustrates the laboratory procedure for the regeneration and purification of the adsorbent material composed of AC impregnated with MgO.

#### Water-based extraction for adsorbent regeneration

**Fig. 2:**
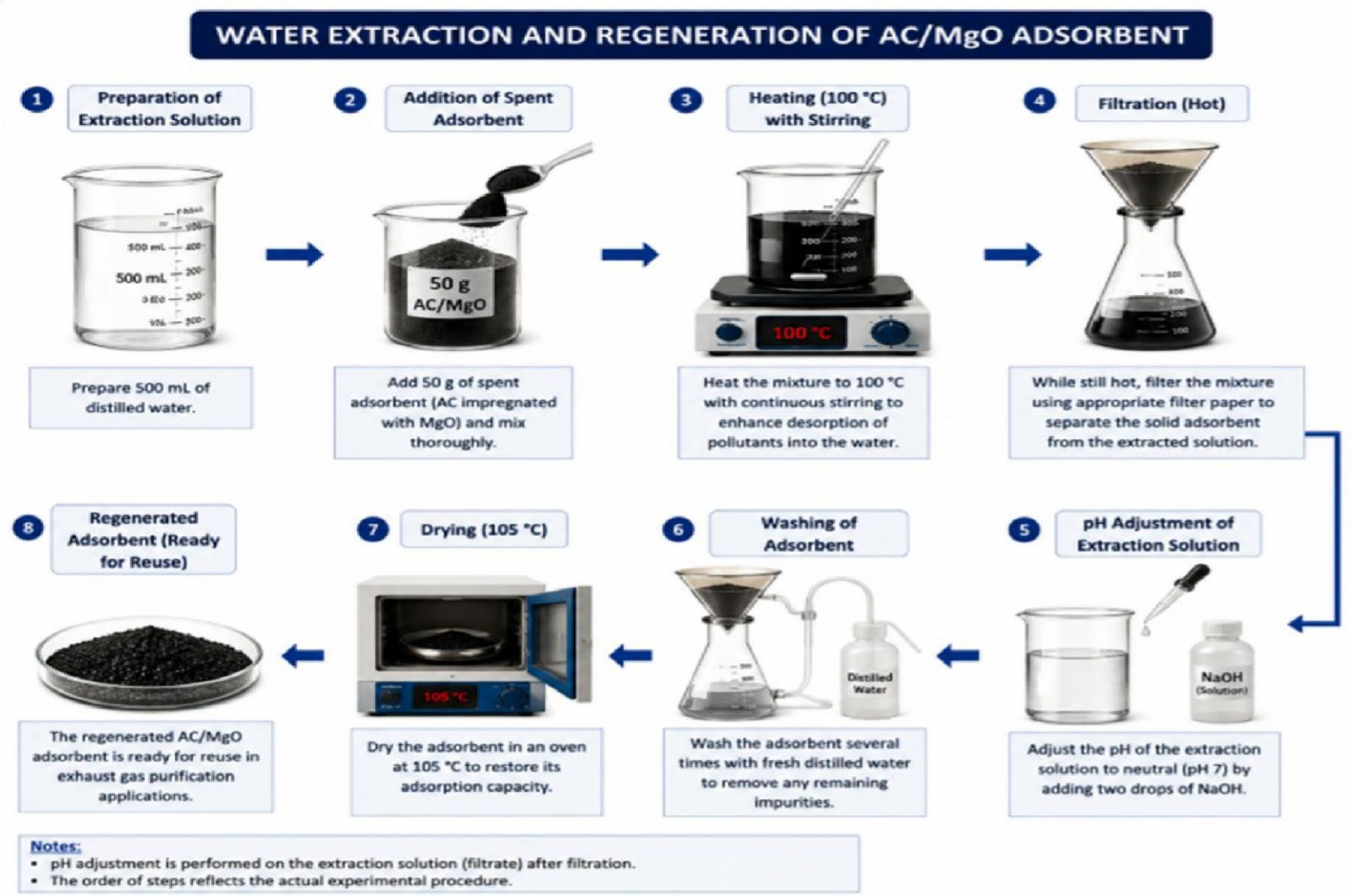
Steps of extraction using H_2_O and activation of activated carbon: laboratory procedure.

The extraction and regeneration process of the spent adsorbent material involved several key steps. First, a 500 mL solution of distilled water was prepared, and 50 g of the spent adsorbent (AC impregnated with MgO) were added and thoroughly mixed. The mixture was then heated to 100 °C using a magnetic stirrer with a heating plate to facilitate the desorption of pollutants from the adsorbent surface into the water. While still hot, the solution was filtered using appropriate filter paper to separate the solid adsorbent from the extracted solution containing the desorbed substances. The pH of the extraction solution was adjusted to a neutral level (pH 7) by adding two drops of sodium hydroxide (NaOH) to ensure stability and prevent undesirable side reactions. The activated carbon was then washed multiple times with fresh distilled water to remove any remaining impurities and ensure complete regeneration. Finally, the adsorbent material was dried in an oven at an appropriate temperature to restore its adsorption capacity, making it suitable for reuse in exhaust gas purification applications. Figure 2. illustrates the laboratory procedure for the regeneration and purification of the adsorbent material composed of AC impregnated with MgO.

#### Acidic extraction for adsorbent regeneration

**Fig. 3:**
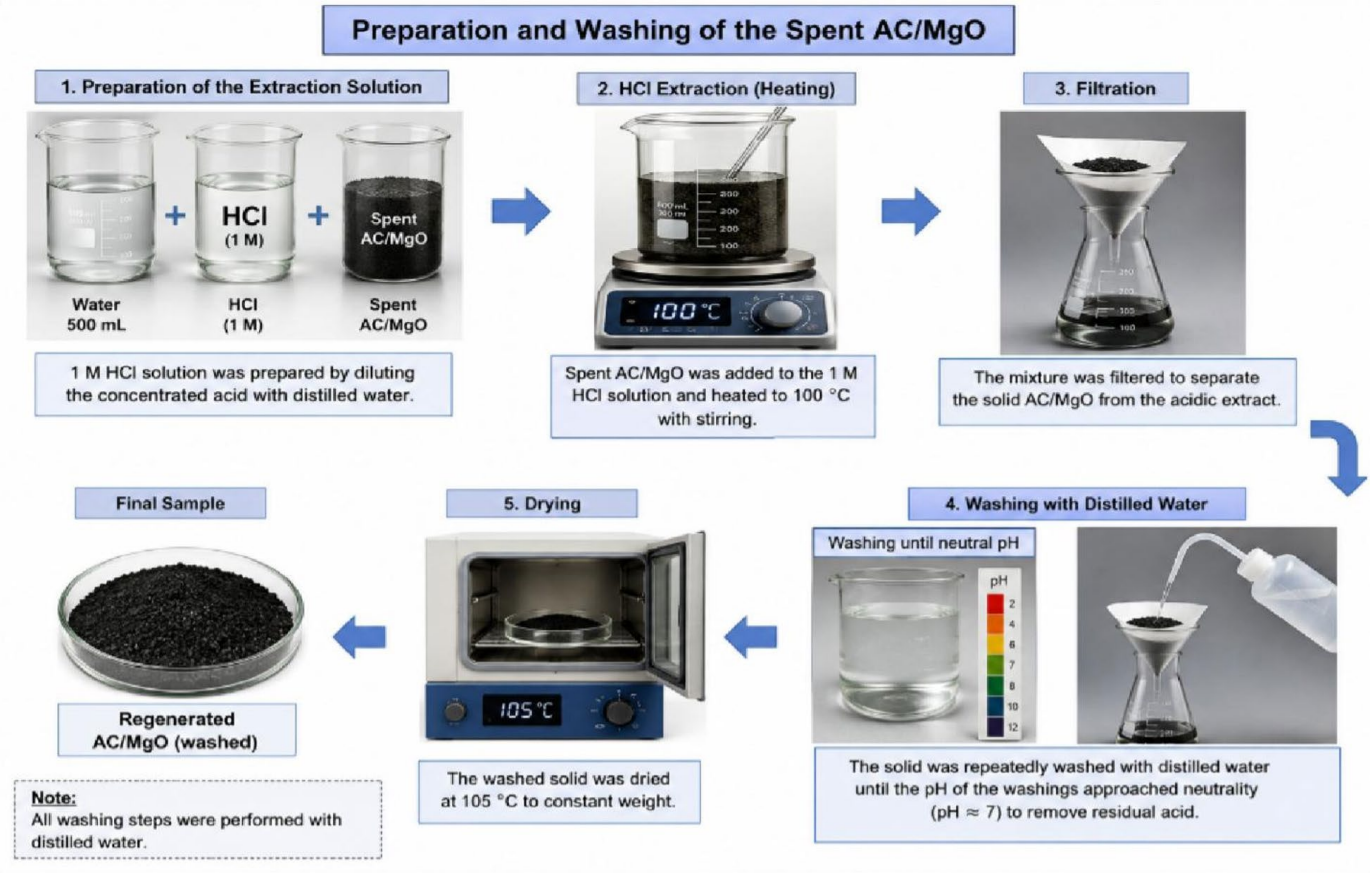
Steps of extraction using HCL and activation of activated carbon: laboratory procedure.

The extraction process involved the use of a diluted hydrochloric acid (HCl) solution to recover elements from the spent adsorbent material. A 500 mL extraction solution was prepared by adding 32 mL of concentrated HCl to distilled water. The resulting diluted acid solution was then used for the extraction process^14^. Subsequently, 50 g of spent adsorbent (AC impregnated with MgO) was added to the prepared HCl solution and thoroughly mixed. The mixture was heated to 100 °C under continuous stirring using a magnetic stirrer to enhance the dissolution of adsorbed elements into the acidic solution. After extraction, the suspension was filtered to separate the solid adsorbent from the liquid extract containing the recovered elements. The recovered AC was washed several times with distilled water to remove any residual acid and ensure complete purification. The pH of the liquid extract was subsequently adjusted by the gradual addition of acetic acid (CH₃COOH) until it reached pH 7. The washed carbon was then dried in an oven at 105 °C for 3 h to restore its adsorption properties, making it suitable for reuse in exhaust gas purification applications. Figure 3 illustrates the laboratory procedure for the regeneration and purification of the spent AC impregnated with MgO.

### Characterization of reactivated carbon and nutrients

#### Reactivation of activated carbon

Reactivated carbon is spent AC that undergoes regeneration to restore its adsorptive properties, providing a cost-effective and environmentally sustainable alternative to virgin carbon^15^. Among the reactivation methods are thermal treatment, where carbon is heated at 600–900 °C in a non-oxidizing atmosphere to remove impurities; chemical reactivation, which involves acid washing (e.g., HCl, HNO₃) to enhance impurity removal; and steam reactivation, where steam desorbs volatile compounds, commonly used in solvent recovery^16^.The spent AC was reactivated through three separate washing processes: acid washing using HCl at a concentration of 1 M, water washing with distilled water, and alkaline washing using a 3% KOH solution. Each treatment aims to remove impurities and restore the adsorption capacity of the carbon.To evaluate the efficiency of reactivation, the regenerated carbon from each washing method will undergo a series of tests, including methylene blue number to assess adsorption performance, iodine number as an indicator of surface area and microporosity, ash content to determine residual inorganic matter, pH measurement, and elemental analysis of C, N, S and content Ash to examine the chemical composition.Additionally, before the purification process, a thorough examination was carried out on carbon samples impregnated with magnesium oxide and samples that had been cleaned with KOH, H2O, and HCl solutions to examine the impact of these treatments on the carbon’s characteristics. ISOTHERMAL analysis was used to examine adsorption behavior, NLDFT modeling was used to assess micropore and mesopore distributions, and BET and BJH measurements were used to ascertain the surface area and pore size distribution, respectively. Furthermore, FTIR spectroscopy was utilized to detect the functional groups on the carbon surface, and XRD analysis was utilized to describe the reactivated carbon’s crystalline structure and phase composition. BET surface area, pore volume, and elemental composition analyses were conducted as single analytical measurements. Therefore, standard deviations, error bars, and statistical comparisons among treatments were not determined for these parameters.

#### Evaluation of recovered elements for sustainable agricultural fertilization

To assess the potential recovery of nutrients from the spent activated carbon used in exhaust gas purification, the three extracted solutions obtained from acidic, alkaline, and water-based washing processes will be subjected to chemical analysis. The concentrations of potassium (K), magnesium (Mg), and iron (Fe) were quantified using appropriate analytical techniques. These elements were selected due to their agronomic significance:

- Potassium (K): Regulates osmotic balance, enzymatic activation, and enhances plant stress tolerance^17^.
- Magnesium (Mg): An essential component of chlorophyll, playing a critical role in photosynthesis and enzymatic functions^18^.
- Sulfates (SO₄²⁻): A vital sulfur source required for amino acid synthesis and metabolic processes^19^.
- Nitrates (NO₃⁻): A primary nitrogen source, essential for protein synthesis and chlorophyll formation^20^.
- Iron (Fe): Facilitates electron transfer in photosynthesis and is a cofactor in various enzymatic reactions^21^.

The obtained data will provide insights into the feasibility of utilizing these extracted solutions as a potential nutrient source for sustainable agricultural fertilization.

#### Applications of the washing processes

##### Activated carbon

Activated carbon reactivated through three different washing methods (distilled water, acidic solution with HCl, and alkaline solution with 3% KOH) was used to treat irrigation water artificially contaminated with two commonly used soil-applied pesticides: chlorpyrifos and atrazine.

##### Effluent washing solutions – use in germination testing

In addition to the use of reactivated carbon itself, the washing effluents resulting from the three reactivation processes (water, acidic, and alkaline washes) were collected and used to irrigate barley seeds. These solutions may contain nutrients or compounds released during the reactivation treatments. The treated washwaters were applied to sterilized Petri dishes lined with moistened cotton and evenly seeded with barley grains. The dishes were incubated under controlled conditions (constant temperature and partial darkness) for a period of 7 days. Germination rate and seedling length were recorded as indicators of the stimulatory or inhibitory effects of the washing solutions on plant development. This experiment aims to assess the potential use of such effluents as a nutrient-rich medium or biostimulant in sustainable agricultural systems.

##### Phytotoxicity assessment and enzymatic activity analysis

The phytotoxicity of washing effluents was assessed by a seed germination bioassay, following the Zucconi test^22^. We germinated barley seeds (Hordeum vulgare) of the same size and weight on Petri plates under controlled conditions (25 °C, continuous light) and watered them with varying amounts (1, 2, and 3 mL) of washing solutions. We used tap water as a control. We measured germination percentage, shoot length, and root elongation and compared them to the control^23^. To facilitate the phytotoxicity evaluation, the activities of essential hydrolytic enzymes implicated in seed reserve mobilisation were assessed in germinated seeds. We measured α-amylase activity using the method given^24^, protease activity using the method provided by^25^, and lipase activity using the titrimetric method described^26^. We measured enzyme activity in units per gramme of fresh weight and utilised them as biochemical markers to assess if the washing effluents helped or hurt the growth of early seedlings. To enhance clarity and maintain uniform terminology across the text, Table 1 encapsulates the coding system and treatment history of the activated carbon samples used in this research.

**Table 1:** Sample coding and description of the activated carbon materials used in this study.

Sample code	Description
Spent AC/MgO	MgO-impregnated activated carbon collected after gas filtration and before regeneration
H₂O-AC/MgO	Spent AC/MgO regenerated by water washing
KOH-AC/MgO	Spent AC/MgO regenerated by KOH washing
HCl-AC/MgO	Spent AC/MgO regenerated by HCl washing

The sample codes presented in Table X are used consistently throughout the manuscript to distinguish between the untreated spent adsorbent and the regenerated materials obtained after different washing treatments.

## Results & discussion section

### Activated carbon efficiency test

#### Washing effects on activated carbon

AC derived from coconut shells typically has an ash content ranging between 3% and 5%, making it suitable for applications that require high purity, such as gas purification^27^. The effect of washing agents on the physicochemical characteristics of the activated carbon samples was evaluated. The solid sample washed with KOH (Sample A) exhibited the highest carbon content (89.50%), compared to the samples washed with H_2_O (Sample B: 85.66%) and HCl (Sample C: 80.96%). These analyses, performed at the Central Laboratory Network (CLN) of the National Research Center, showed that this enhancement can be attributed to the strong alkaline properties of KOH, which effectively remove organic impurities without significantly altering the carbon matrix. Although Sample A showed the highest ash content (1.897%), it remained within an acceptable range for adsorption applications, making it the most suitable candidate for reuse in gas purification systems. On the other hand, Sample C presented the lowest ash content (1.654%), indicating the efficacy of HCl in removing inorganic residues; however, its lower carbon content may limit its adsorption efficiency. The type of washing agent used significantly influences the composition of activated carbon, as indicated by the results. Despite a modest rise in ash content, KOH treatment improves adsorption performance by efficiently eliminating organic contaminants without harming the carbon structure. HCl, on the other hand, is better at eliminating inorganic residues, which lowers the ash concentration but also the carbon content, which may restrict the effectiveness of adsorption. When compared to chemical treatments, the effects of water cleaning are moderate^28^.

#### Surface and porosity of treated AC

The porosity properties of the activated carbon (AC) samples were examined using BET surface area analysis, nitrogen adsorption-desorption isotherm analysis, pore size distribution analysis using the BJH method, and NLDFT analysis. Three regenerated activated carbon samples obtained using KOH, H₂O, and HCl washing treatments were used for these analyses. All measurements were carried out at the National Research Center’s Center of Excellence for Medical Research (NRC).

##### The brunauer–emmett–teller (BET)

**Fig. 4:**
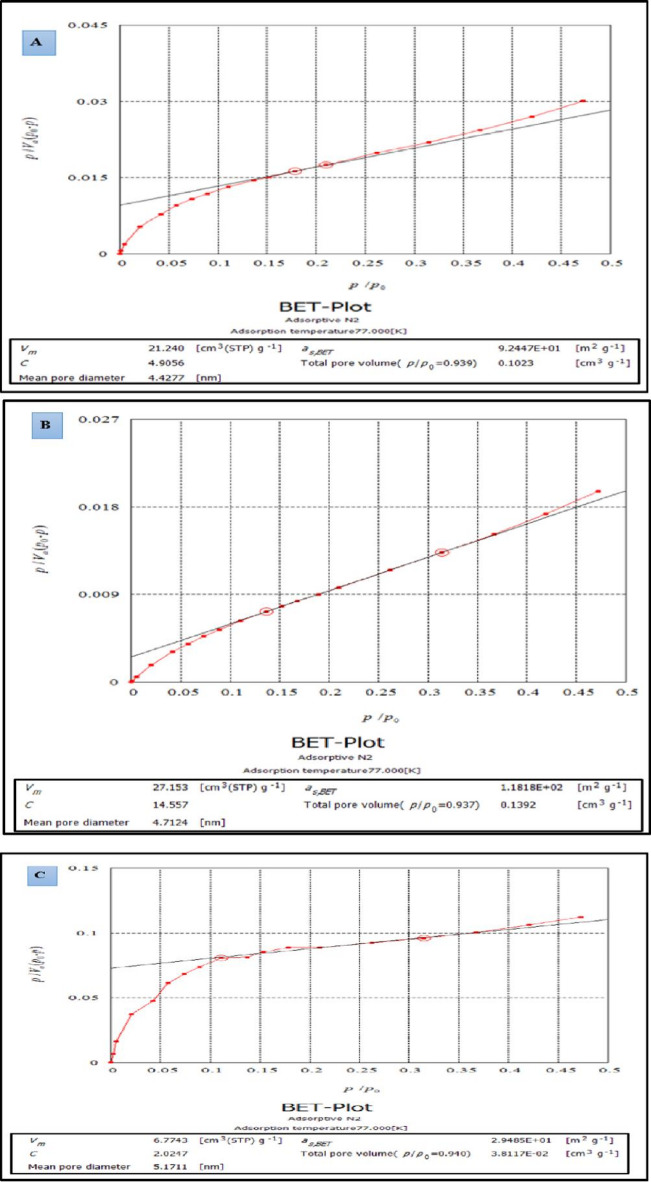
AC samples washed with A: KOH, B: H₂O, and C: HCl were evaluated for porosity using BET.

BET surface area analysis was used to assess how various washing procedures affected the textural characteristics of the AC after gas filtration. An untreated spent AC/MgO sample was used as a control reference for comparison with the regenerated samples. Total pore volume, mean pore diameter, BET surface area, and BET C constant for the untreated spent, KOH-washed, H₂O-washed, and HCl-washed samples are summarized in Table 2. While the corresponding nitrogen adsorption-desorption isotherms are presented in Fig. 4. Understanding how each treatment affects porosity and surface properties that are essential for adsorption performance is possible thanks to these factors.

**Table 2:** Textural properties of AC samples after different washing treatments (BET analysis).

Washing agent	BET surface area (m²/g)	Total pore volume (cm³/g)	Mean pore diameter (nm)
KOH	92.45	0.1023	4.43
H2O	118.18	0.1392	4.71
HCL	29.49	0.0381	5.17

The untreated spent AC/MgO sample was used as a control reference for comparison with the regenerated samples.The BET surface area (118.18 m²/g) and total pore volume (0.1392 cm³/g) of the sample that was washed with distilled H_2_O were the highest, suggesting that H_2_O successfully increased pore accessibility without compromising the carbon structure. A surface area of 92.45 m²/g and a pore volume of 0.1023 cm³/g were also improved in the KOH-washed sample, indicating effective organic residue removal. In contrast, the HCl-washed sample showed a significantly lower pore volume (0.0381 cm³/g) and surface area (29.49 m²/g), which could be related to acidic treatment-induced pore blockage or structural deterioration. Considering the potential effects of HCl, our findings indicate that water is the best washing agent for maintaining and improving the porosity of AC. The findings indicate that, whereas HCl may result in pore blockage and reduced pore accessibility, reducing surface area and pore volume, distilled water maintains and improves pore structure by eliminating contaminants without harming the carbon matrix^28^.

##### Nitrogen adsorption-desorption isotherm analysis

**Fig. 5:**
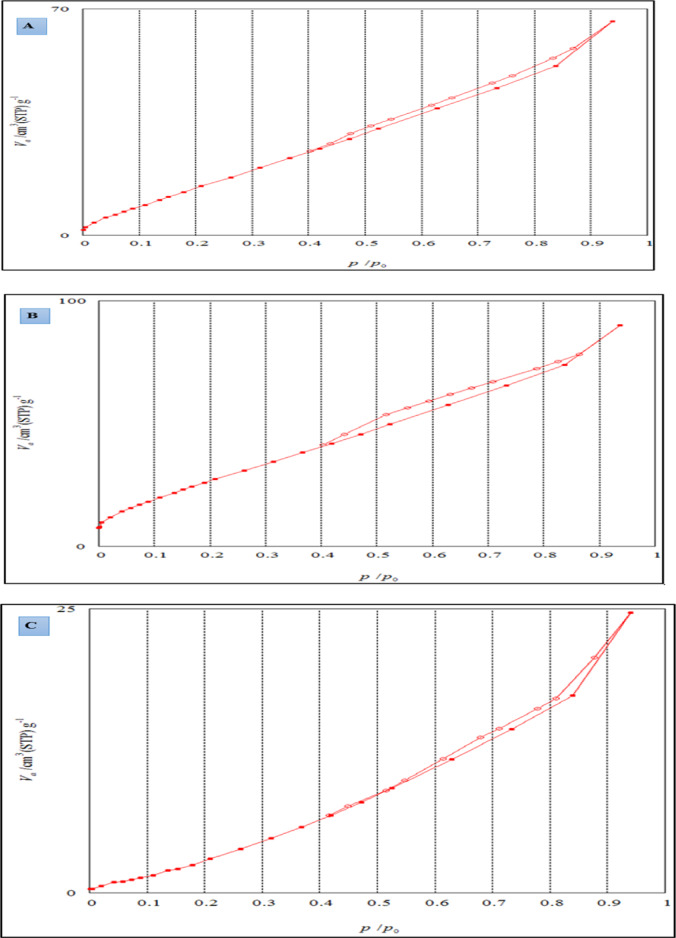
AC samples washed with A: KOH, B: H₂O, and C: HCl were evaluated for isotherms.

At 77 K and a saturation pressure of 108 kPa, nitrogen adsorption–desorption isotherms were recorded for samples washed with KOH, H₂O, and HCl in order to assess the impact of various washing procedures on the porosity of AC impregnated with MgO. The KOH-washed sample exhibited a Type IV isotherm with an H4 hysteresis loop, indicating the presence of micro- and mesoporous structures. with a clear H4-type hysteresis loop that the KOH-washed sample displayed, as seen in Fig. 5. the H_2_O sample had a Type II isotherm that reflected multilayer adsorption on a relatively accessible surface and lacked obvious hysteresis. In contrast, the HCl-washed sample showed a nearly flat isotherm and little nitrogen uptake, indicating possible structural deterioration or pore blockage brought on by the acid treatment. Table 3 presents a comparison of the isotherm behavior for the AC samples.

**Table 3:** Comparison of isotherm behavior for AC samples.

Washing agent	BJH surface area (m²/g)	Hysteresis loop	Interpretation
KOH	Type IV	Present (H4-type)	Developed micro- and mesoporous structure; improved pore accessibility and adsorption performance
H2O	Type II	Absent	Limited pore enhancement; weak surface interaction
HCL	Flattened IV	Absent	Possible pore blockage and reduced pore accessibility; minimal porosity and adsorption performance

The IUPAC categorisation of the H₂O-washed sample showed a Type II adsorption isotherm, which is associated with multilayer adsorption on a generally accessible surface without a clear hysteresis loop. This behaviour indicates lower pore development than the KOH-washed sample, which showed a Type IV isotherm with an H₄ hysteresis loop typical of mesoporous and microporous structures.

According to the results, the final porosity and adsorption efficiency of AC are mostly determined by the post-treatment washing agent. The interior pore network and adsorption properties are significantly improved by alkaline washing with KOH, as seen by the isotherm shape and noticeable hysteresis. H_2_O washing offers very little improvement, whereas HCl treatment may reduce pore accessibility and adversely affect the pore structure.In applications that demand high surface area and stable mesoporous networks, acidic treatment was less effective under the conditions evaluated in this study; alkaline treatment is ideal for activating and maintaining the porous architecture. The washing agent significantly influences the porosity and adsorption performance of activated carbon, as these results validate. While acid treatment (HCl) may reduce pore accessibility and adsorption performance, alkaline treatment (KOH) improves pore development and stability^29^.

##### Barrett-Joyner-Halenda (BJH)

**Fig. 6:**
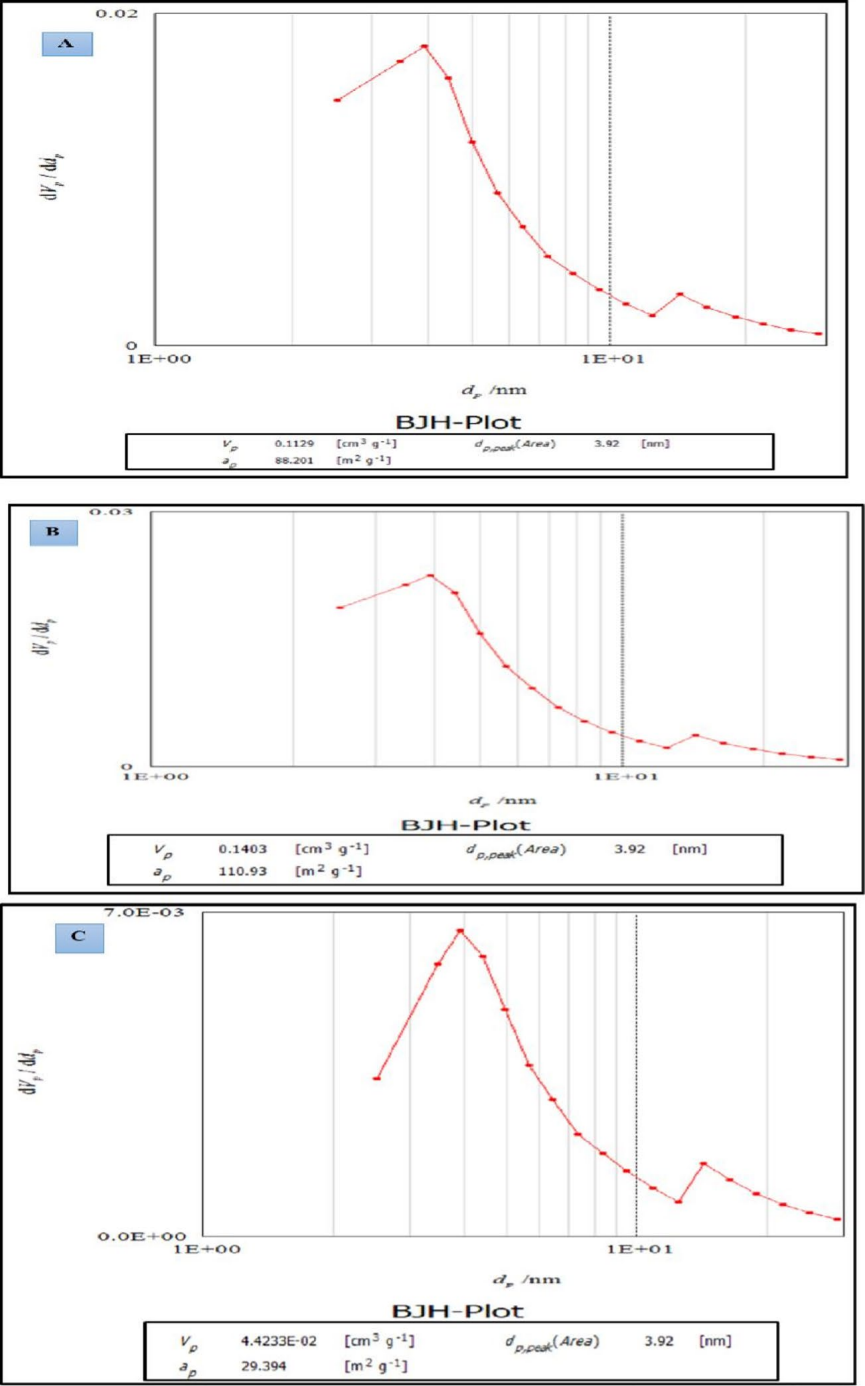
AC samples washed with A: KOH, B: H₂O, and C: HCl were evaluated for BJH.

Significant variations in the textural characteristics of three AC samples that had been cleaned with KOH, H₂O, and HCl were found by BJH analysis. Indicating superior pore formation, the H_2_O washed sample had the largest total pore volume (0.1403 cm³/g) and specific surface area (110.93 m²/g). With a surface area of 88.20 m²/g and a pore volume of 0.1129 cm³/g, the KOH-washed sample had a mesoporous structure that was ascribed to improved pore formation through chemical activation. On the other hand, the HCl-treated sample had a much smaller pore volume (0.0442 cm³/g) and surface area (29.4 m²/g), which may be attributed to pore blockage and reduced pore accessibility following acid treatment. Table 4 provides a summary of these findings and the pore size distribution curves displayed in Fig. 6.

**Table 4:** Textural properties of AC samples based on washing method.

Washing agent	BJH surface area (m²/g)	Total pore volume (cm³/g)	Porosity type
KOH	**88.20**	**0.1129**	**Mesoporous**
H2O	**110.93**	**0.1403**	**Mesoporous**
HCL	**29.40**	**0.0442**	**Moderate Surface Area**

It should be noted that the surface area values reported in Table 3 were derived from BJH analysis and therefore differ from the BET surface area values presented in Table 1.

Primarily, the type of washing treatment used affects the variations in the textural characteristics of the AC samples. Because KOH washing chemically activates the carbon structure, it increases porosity by increasing pore volume and surface area. By eliminating soluble contaminants and enhancing pore accessibility, H_2_O washing, as a physical technique, gives the samples their largest surface area. The removal of inorganic residues by HCl washing, on the other hand, may result in pore blockage and reduced pore accessibility, which would decrease the overall pore volume and surface area. These findings show that washing treatment has a significant impact on AC textural characteristics. While H2O increases surface area by eliminating soluble contaminants without causing structural harm, KOH increases porosity through chemical activation. HCl, on the other hand, may reduce pore accessibility because it removes inorganic components from the carbon matrix, which reduces pore volume and surface area^29^.

BJH analysis was used to evaluate mesopore size distribution, whereas NLDFT analysis was applied to characterize the microporous structure of the activated carbon. The combined use of both methods provided a comprehensive assessment of pore characteristics following regeneration treatments.

##### Non-local density functional theory (NLDFT)

**Fig. 7:**
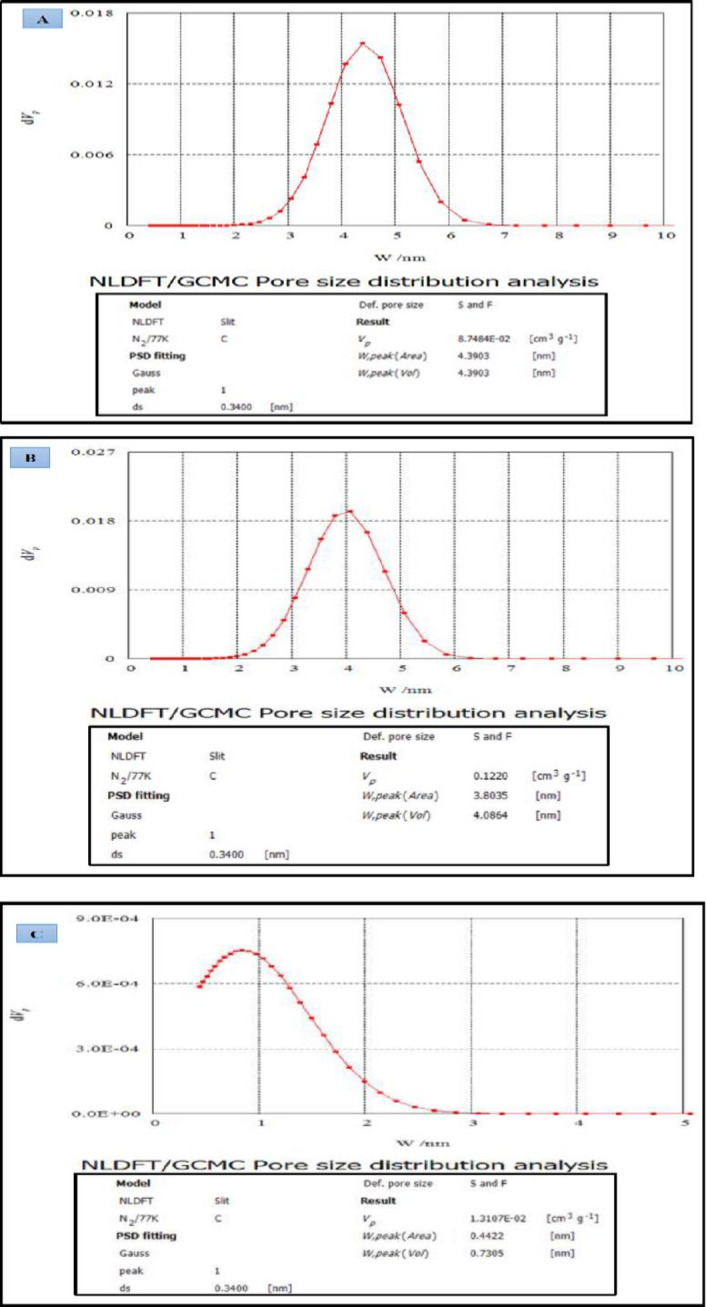
AC samples washed with A: KOH, B: H₂O, and C: HCl were evaluated for NLDFT.

The adsorption capabilities of the AC samples are directly impacted by the observed changes in pore volume and structure. Because of their mesoporous nature and increased pore volume, samples KOH and H₂O may be more accessible to larger adsorbate molecules, which makes them appropriate for uses such as gas storage, catalysis, and the removal of large impurities. The KOH treatment promoted the development of a well-defined porous network through chemical activation, while water washing (H₂O) helps to preserve and possibly enlarge existing pores. Conversely, the smaller pore volume and lower pore accessibility, resulting from HCl washing, indicates a more compact pore structure with limited pore volume. This pore structure may favor the adsorption of smaller molecules and gases due to stronger adsorbate-adsorbent interactions within narrow pores but may restrict diffusion rates for larger species. Therefore, the choice of post-treatment significantly tailors the pore characteristics and, consequently, the suitability of the activated carbon for specific adsorption applications. Figure 7.

The results obtained demonstrate that the pore structure and adsorption characteristics of the AC samples are greatly influenced by the washing technique. Because of chemical activation, the KOH-washed sample exhibits a well-developed mesoporous structure with excellent surface area and pore volume, making it a promising option for adsorption applications. The water-washed sample exhibits modest adsorption effectiveness but the largest surface area and pore volume, indicating intact pore structure. The HCl-washed sample, on the other hand, exhibited reduced porosity and pore accessibility, which may limit its adsorption performance. Therefore, HCl washing was less effective in preserving the adsorption-related properties of the carbon, whereas KOH treatment optimally increases it^27^. The NLDFT quantitative parameters acquired for the regenerated AC/MgO samples are summarized in Table 5. The results revealed that the washing treatment had a substantial effect on the pore volume and pore-size distribution, and the H₂O and KOH treatments had more developed pore structures than the HCl treatment. These results confirm the BET analysis and show the critical effect of the regeneration conditions on the final pore properties of AC.

**Table 5:** Comparison of NLDFT-derived pore structure parameters of AC samples.

Washing agent	Vp (cm³/g)	Peak pore width (nm)	Interpretation
KOH	**0.0875**	**4.3903**	**Developed mesoporous structure**
H2O	**0.1220**	**4.0864**	**Highest pore volume**,** mesoporous structure**
HCL	**0.0131**	**0.7305**	**Strong reduction in pore volume**

The significant alterations in pore structure across the washing protocols were confirmed using quantitative NLDFT analysis. The highest pore volume (0.1220 cm³ g⁻¹) was achieved with H₂O treatment, succeeded by KOH (0.0875 cm³ g⁻¹), whereas HCl treatment resulted in a significantly reduced pore volume (0.0131 cm³ g⁻¹). The maximum pore diameters measured approximately 4.09 nm for H₂O and 4.39 nm for KOH, indicating mesoporous configurations. The sample treated with HCl demonstrated a pore-size distribution centered at 0.73 nm with a substantially lower pore volume.

### Nutrient recovery from washing filtrates

**Fig. 8:**
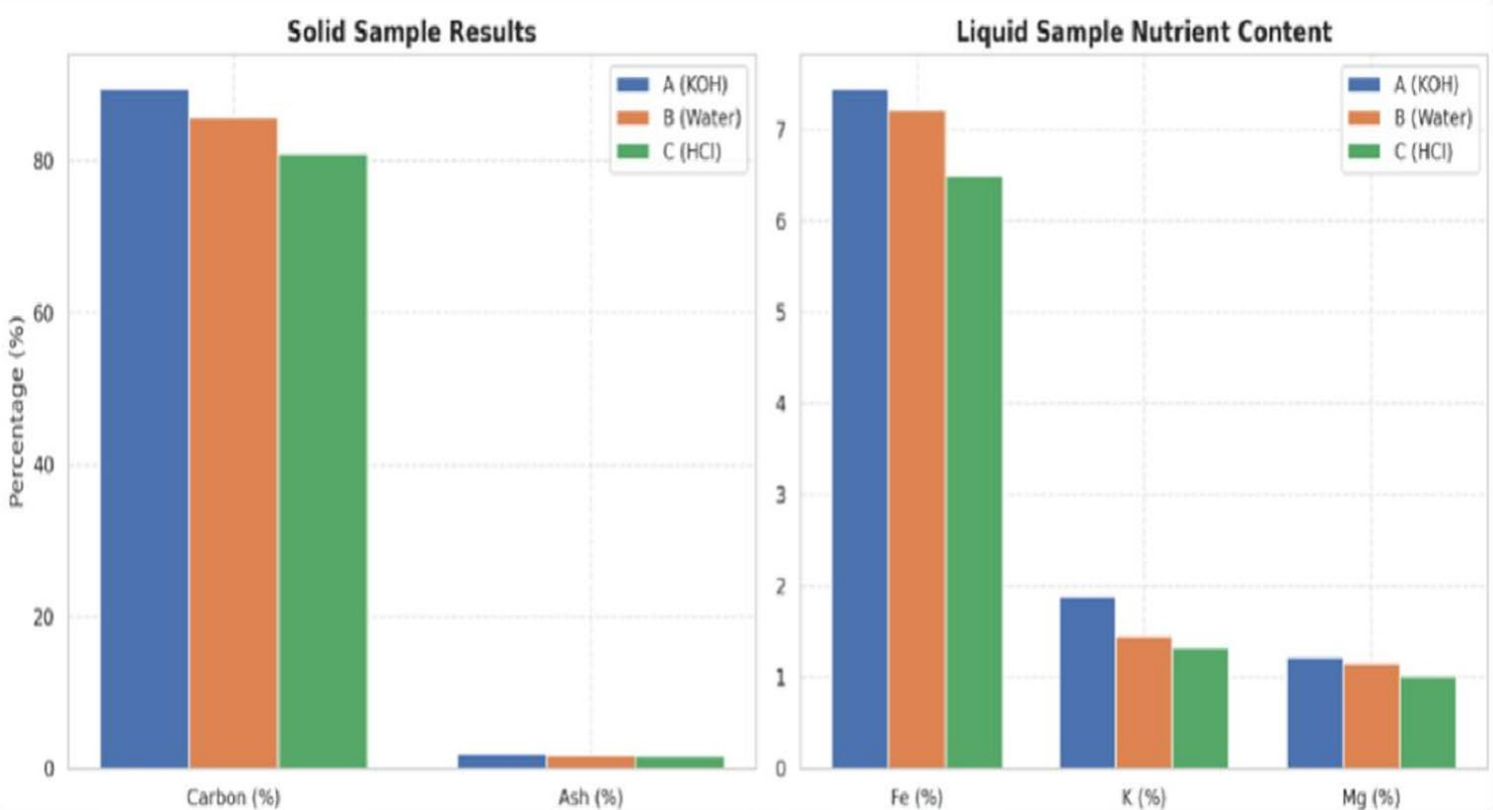
Chemical comparison of AC and solution phases of washed activated carbon.

Regarding the liquid filtrates obtained after washing, KOH demonstrated the highest concentrations of iron (7.45%), potassium (1.89%), and magnesium (1.22%). These nutrients were measured to evaluate their potential for agricultural use. These analyses, performed at the Central Laboratory Network (CLN) of the National Research Center, The nutrient concentrations highlight the potential of this washing solution for agricultural applications; however, further evaluation is required before fertilizer use. This dual benefit—enhancing the solid adsorbent’s performance while recovering nutrient-rich liquid—supports the integration of such processes within circular economy frameworks and sustainable agriculture practices. Table 6 shows the effect of the washing agent on the chemical properties of solid and liquid samples from activated carbon. Figure 8 shows a comparison of the chemical properties of activated carbon samples after washing with alternatives (KOH, H_2_O, HCl) and the concentration of nutrients in the resulting washing solutions.

**Table 6:** Effect of washing agent on chemical properties of solid and liquid samples from activated carbon.

Washing agent	Activated carbon				Solution			Recommended use
	C%	*N*%	S%	Total Ash (%)	Fe %	K%	Mg%	
KOH	89.50	2.03	2.95	1.897	7.45	1.89	1.22	Reuse + Liquid Fertilizer
H2O	85.66	1.56	2.56	1.765	7.22	0.0259	1.15	Balanced Option
HCL	80.96	1.44	2.06	1.654	6.50	0.03	1.01	High Purity (Low Ash)

In the KOH-treated extract, the high potassium concentration may be in part due to the KOH reagent employed during the regeneration process. Therefore, the observed potassium concentration should be interpreted as the total potassium present in the extract, i.e., the recovered potassium plus any potassium contributed by the regeneration reagent. Previous studies on the same AC/MgO adsorbent reported the accumulation of nitrate, sulfate, and carbonate species following diesel exhaust treatment, indicating the successful capture of nutrient-related compounds prior to regeneration^9^. However, because the initial and final masses of individual nutrient species were not quantified during the regeneration experiments, nutrient recovery efficiencies, carbon yield, and complete material balance calculations could not be accurately determined. Future work should include detailed mass balance assessments to quantify nutrient recovery and regeneration efficiency. The environmental safety of the recovered solutions and regenerated AC/MgO **is** an important consideration for their potential future applications. Since the spent activated carbon originated from gas purification filters, potential accumulation of inorganic contaminants or heavy metals cannot be completely excluded. Therefore, although Fe, K, and Mg were evaluated in this study, further comprehensive screening of heavy metals and other contaminants is required before large-scale agricultural or environmental applications.

Iron concentrations were determined using Atomic Absorption Spectroscopy (AAS), and the values are reported as percentages (%) according to the analytical laboratory report. The relatively high Fe content observed in the recovered solutions (6.50–7.45%) may be attributed to the release of retained iron-containing species and inorganic deposits during the regeneration process. captured by the activated carbon during gas filtration. During the regeneration process, part of these retained iron species may be released into the extraction solution, resulting in elevated Fe concentrations. Activated carbon is known to retain metal ions within its porous structure, which promotes the retention of iron within its porous structure. Although iron is an essential micronutrient, its impact on applications such as germination depends mainly on its bioavailability rather than its total concentration^30^. The iron level in the activated carbon samples is attributable to the natural mineral composition of the biomass precursor and the subsequent accumulation of inorganic residues following carbonization and activation procedures. Activated carbon generated from biomass generally retains residual metals, such as iron, in the form of mineral matter, which may either persist within the carbon matrix or become concentrated following the elimination of volatile components. The porous structure and elevated surface area of activated carbon enhance the adsorption and redistribution of metal ions throughout processing and washing stages^31^.

### Effect of different washing treatments on AC/MgO properties

The textural properties of AC/MgO after reactivation by different washing treatments are summarized in Table 7. H_2_O washing produced the highest BET surface area and total pore volume, indicating superior preservation and accessibility of the pore structure. KOH washing also significantly improved the textural properties compared with the untreated sample and promoted the development of microporous structures, which may enhance adsorption performance. In contrast, HCl washing led to a decrease in surface area and pore volume. The acidic medium effectively dissolved some metallic oxides; however, it may also have reduced pore accessibility and pore volume, thereby reducing the number of available adsorption sites. Therefore, H₂O washing was the most effective treatment for preserving surface area and pore volume, whereas KOH washing provided additional benefits in nutrient recovery and pore development.

**Table 7:** Comparison of AC/MgO properties after different washing treatments.

Sample	BET surface area	Total pore volume	Pore structure	Main observation
H₂O washed	Highest among samples	Highest increase	Well-developed micro- and mesoporous structure	Best preservation of pore structure
KOH washed	Significant increase	Moderate increase	Developed micro- and mesoporous structure	Enhanced micropore development and nutrient recovery
HCl washed	Lowest	Decreased	Limited porosity	Possible pore blockage and reduced pore accessibility

The washing agent process explains the differences in pore structure. Distilled water removes mostly soluble salts, loosely bound impurities, and deposited contaminants from the pore network without changing the carbon structure, enhancing pore accessibility and structural integrity. However, KOH reacts with carbonaceous surfaces and residual deposits, promoting chemical activation and pore opening, which facilitates the development of additional micro- and mesoporous structures. Hydrochloric acid primarily dissolves inorganic deposits and metal oxides retained within the carbon matrix, while excessive acid treatment may decrease pore accessibility and pore development. These mechanisms are in accordance with the BET, BJH, and NLDFT outcomes obtained in this study^32^.

Table 8. illustrates a comparison of the activated carbon sample prior to washing and the regenerated samples subjected to treatment with H₂O, KOH, and HCl. The findings indicate that treatments with H₂O and KOH significantly enhanced surface area, pore volume, and pore accessibility relative to the untreated specimen. Conversely, HCl treatment yielded only limited improvement. These results validate the efficacy of the regeneration procedures in reinstating the textural characteristics of activated carbon, especially for the H₂O and KOH methods^33^.

**Table 8:** Comparison of BET and NLDFT parameters of AC before and after regeneration treatments.

Sample	BET surface area (m²/g)	Total pore volume (cm³/g)	Mean pore diameter (nm)	NLDFT pore volume (cm³/g)	Peak Pore Width (nm)
Fresh AC	24.50	0.0365	3.92	0.0271	4.3903
KOH	88.20	0.1129	3.92	0.0875	4.3903
H₂O	110.93	0.1403	3.92	0.1220	4.0864
HCl	29.39	0.0442	3.92	0.0131	0.7305

Table 8. comparesthe textural characteristics of activated carbon prior to washing and subsequent to regeneration employing various washing methods. Both H₂O and KOH applications improved surface area, pore volume, and pore accessibility relative to the untreated specimen. The H₂O-washed specimen demonstrated the greatest BJH and NLDFT pore volumes, signifying optimal retention of pore architecture. Conversely, HCl treatment yielded merely a marginal enhancement compared to the untreated specimen and exhibited the minimal NLDFT pore volume, indicating limited pore accessibility. The findings indicate that the washing procedure influences the textural properties and pore structure of AC, with H₂O and KOH yielding the most advantageous regeneration results.

### Applications of the washing processes

#### Utilization of washing effluents in barley germination assays

The washing effluents generated from spent AC/MgO were evaluated for their potential impact on early plant development using barley germination assays. This application serves as a sensitive bio indicator for assessing the phytotoxicity or growth-promoting properties of the effluents^34^. By exposing barley seeds to different effluent types—derived from water, KOH, and HCl washing solutions—and applying them at varying concentrations, the study aimed to determine whether residual chemicals or dissolved mineral components influence seed germination and root elongation. This approach not only provides insight into the environmental safety of these washing processes but also offers a practical method for assessing whether the resulting effluents could have potential agricultural applications after further evaluation or require additional treatment before disposal. However, this germination assay represents an initial phytotoxicity screening approach, and further long-term plant growth experiments under greenhouse or field conditions are required to fully evaluate the agronomic performance of the recovered nutrient solutions.

#### Descriptive statistical analysis

Descriptive statistics were performed to summarize variations in barley root elongation across treatments and concentrations. Mean germination lengths showed clear differences among the washing effluents. The H₂O effluent exhibited the highest mean values, indicating a potentially favorable effect on seed growth^35^. The control group showed moderate and consistent germination, serving as a stable baseline.

In contrast, the KOH washing effluent produced the lowest germination lengths across all concentrations, with mean values drastically reduced and in some cases approaching complete inhibition. The HCl effluent demonstrated an intermediate pattern, showing moderate suppression at lower concentrations and more pronounced inhibition at the highest concentration. The variability within replicates was minimal for most treatments, indicating good experimental repeatability and reliable measurement of germination performance.

**Fig. 9:**
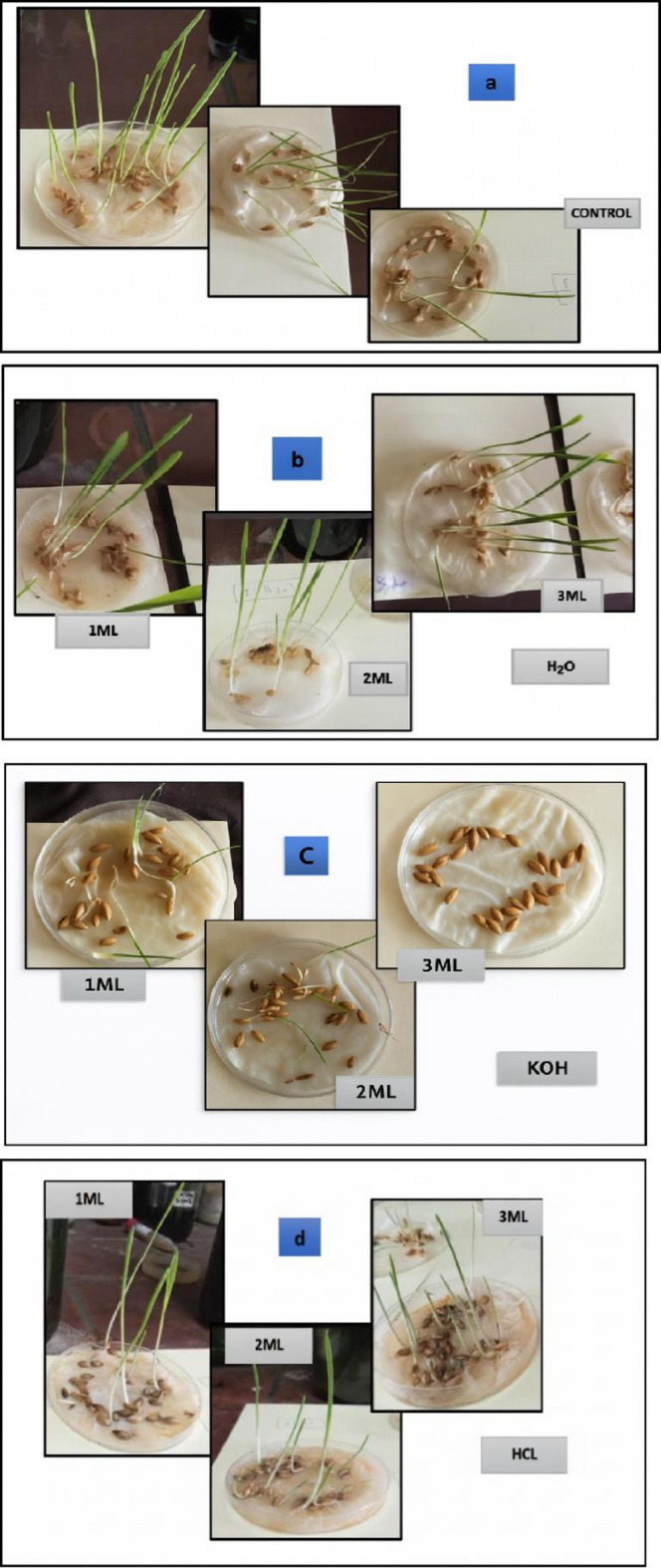
Barley seed germination under different washing effluent treatments (**a**) control, (**b**) H2O, (**c**) KOH and (**d**) HCL. Overall, these descriptive patterns suggested notable differences among treatments even before inferential analysis, supporting the need for formal statistical testing. Figure 9 presents representative images of barley seed germination under the different washing effluent treatments, visually illustrating the variation in root elongation observed across the experimental groups.

The KOH washing effluent caused a marked decrease in germination at all concentrations, probably due to its high alkalinity, which promotes phytotoxic stress and interferes with normal metabolic processes. The HCl effluent demonstrated a very mild impact, sustaining acceptable germination rates at lower concentrations, likely attributable to the less detrimental characteristics of moderate acidity, which may influence seed germination behavior under the tested conditions^36^. Despite the detection of higher iron concentrations (6–7 mg/L) in the washing filtrates, no direct negative effect on germination was noted. This suggests that the detected iron concentrations were not associated with observable phytotoxic effects under the tested conditions. suggesting that germination responses were predominantly influenced by the physicochemical characteristics of the washing agents, especially pH, rather than metal concentration^37^.

#### Interpretation of statistical findings

The one-way ANOVA results demonstrated a highly significant difference among the filtrates obtained from washing magnesium-oxide–loaded activated carbon used in tractor exhaust gas purification. Because the pH of the HCl and KOH washing solutions was adjusted prior to use, the observed variations in barley seed germination cannot be explained solely by acidity or alkalinity. Instead, they reflect differences in the chemical species mobilized from the activated carbon during washing.Filtrates derived from water-washed carbon exhibited the highest germination lengths, surpassing even the control group. This suggests that distilled water primarily removed weakly soluble or neutral residues without introducing substantial chemical stress, resulting in minimal phytotoxicity. In contrast, filtrates from KOH-washed carbon produced the lowest germination values despite pH adjustment. This indicates that alkaline washing released soluble species and dissolved components that may have contributed to increased ionic strength and imposed strong inhibitory effects on germination. Therefore, dilution, pH adjustment, or further treatment of KOH-derived filtrates may be required before their potential agricultural application.

Filtrates from HCl-washed carbon produced intermediate levels of inhibition. Acid washing may solubilize chloride ions and retained metal species that generate a moderate but noticeable reduction in germination, although their toxicity remains lower than that associated with KOH-derived filtrates.Overall, the findings indicate that the chemical composition of the wash water, shaped by the interaction between spent AC/MgO and the washing agent, is the primary determinant of phytotoxicity. Water washing yields the least harmful effluent, HCl washing results in moderate toxicity, and KOH washing produced the inhibitoriest filtrates. These results underscore the importance of selecting washing procedures that minimize the environmental impact of regenerated exhaust-treatment carbon.

A one-way ANOVA was conducted to determine whether the different washing filtrates from MgO-loaded activated carbon significantly affected barley seed germination. The statistical output is summarized in Table 9.

**Table 9:** Biological grouping of treatments based on germination mean values. Treatments with higher germination means were assigned to Group A, whereas lower-performing treatments were assigned to Group B.

Washing solution	Mean ± SD (cm)	Group
H2O_2ml	13.33	A
H2O_1ml	11	A
H2O_3ml	10.93	A
Control	10.33	A
HCL_1ml	9.33	A
HCL_2ml	8.67	A
HCL_3ml	5.33	B
KOH_1ml	5.83	B
KOH_2ml	5.33	B
KOH_3ml	0.83	B

Values are expressed as mean ± SD. Means followed by the same letter are not significantly different at *p* ≤ 0.05 (LSD test).

**Fig. 10:**
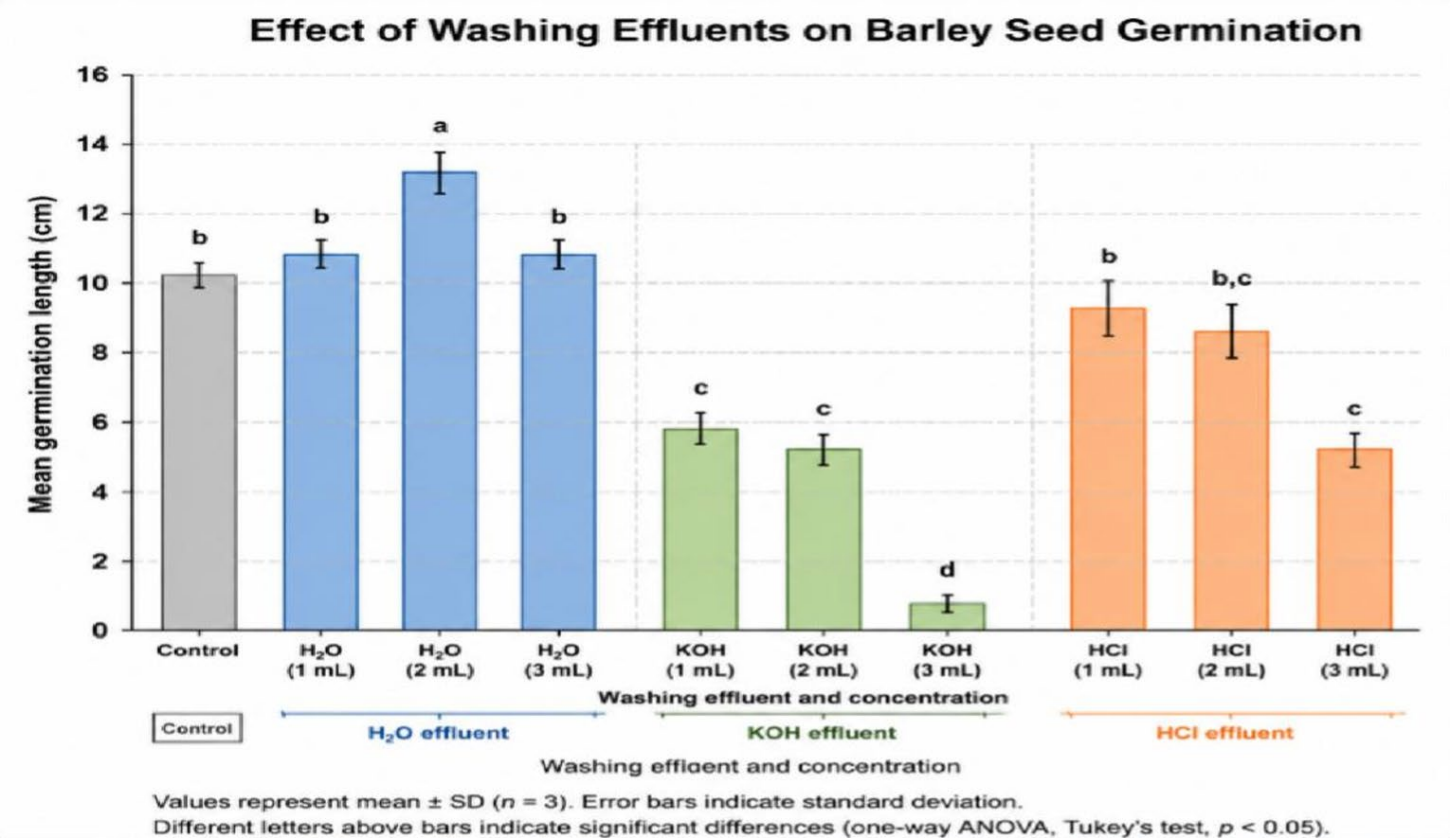
Mean germination length (cm) of barley seeds exposed to filtrates obtained from washing spent AC/MgO using different washing solutions.

Treatments are separated into two biological groups to reflect the overall trend in germination response. Group A includes the control and all water and low-acid filtrates, which exhibited higher germination and lower phytotoxicity. Group B includes KOH and high-acid filtrates, which produced the lowest germination values, indicating stronger inhibitory effects. Figure 10 Shows that water-washed filtrates produced the highest germination, while KOH filtrates resulted in the lowest, with acid washes displaying intermediate values.

## Effect of washing agents on hydrolytic enzyme activities in barley seeds

As presented in Table 10. The activities of protease, lipase, and α-amylase enzymes in barley were significantly influenced by the type and concentration of the washing agent used. Water washing (H₂O) led to a concentration-dependent increase in enzymatic activities. For protease, activity ranged from 180 to 194 U/g FW at 1 ml to 210–219 U/g FW at 3 ml, reflecting an approximate 15% increase at 3 ml compared to 1 ml. Similarly, lipase and α-amylase activities showed enhancements, with protease, lipase, and α-amylase at 3 ml H₂O showing a 10–15% increase compared to the 1 ml treatment, suggesting a moderate activation effect of hydration.

**Table 10:** Effect of washing agents on hydrolytic enzyme activities in barley seeds (mean ± SD, *n* = 3).

Treatment	Protease (U g⁻¹ FW)	Lipase (U g⁻¹ FW)	α-Amylase (mg maltose g⁻¹ FW)
Control	199.3 ± 12.1 a	158.0 ± 9.2 a	0.80 ± 0.07 a
H2O (1 ml)	182.7 ± 10.2 ab	135.0 ± 5.6 b	0.81 ± 0.04 a
H2O (1 ml)	194.0 ± 4.0 a	161.0 ± 3.0 a	0.88 ± 0.02 a
H2O (3 ml)	214.3 ± 4.5 a	168.0 ± 1.0 a	0.92 ± 0.04 a
Control	199.3 ± 12.1 a	158.0 ± 9.2 a	0.80 ± 0.07 a
HCL (1 ml)	162.0 ± 3.5 b	151.0 ± 9.5 a	0.74 ± 0.06 b
HCL (2 ml)	188.0 ± 4.0 ab	163.3 ± 5.0 a	0.83 ± 0.06 a
HCL (3 ml)	206.3 ± 5.0 a	168.0 ± 3.5 a	0.85 ± 0.04 a
Control	199.3 ± 12.1 a	158.0 ± 9.2 a	0.80 ± 0.07 a
KOH (1 ml)	56.0 ± 13.6 c	53.3 ± 4.0 c	0.31 ± 0.05 c
KOH (2 ml)	47.3 ± 4.7 c	43.0 ± 7.5 c	0.24 ± 0.03 c
KOH (3 ml)	ND	ND	ND

**Fig. 11:**
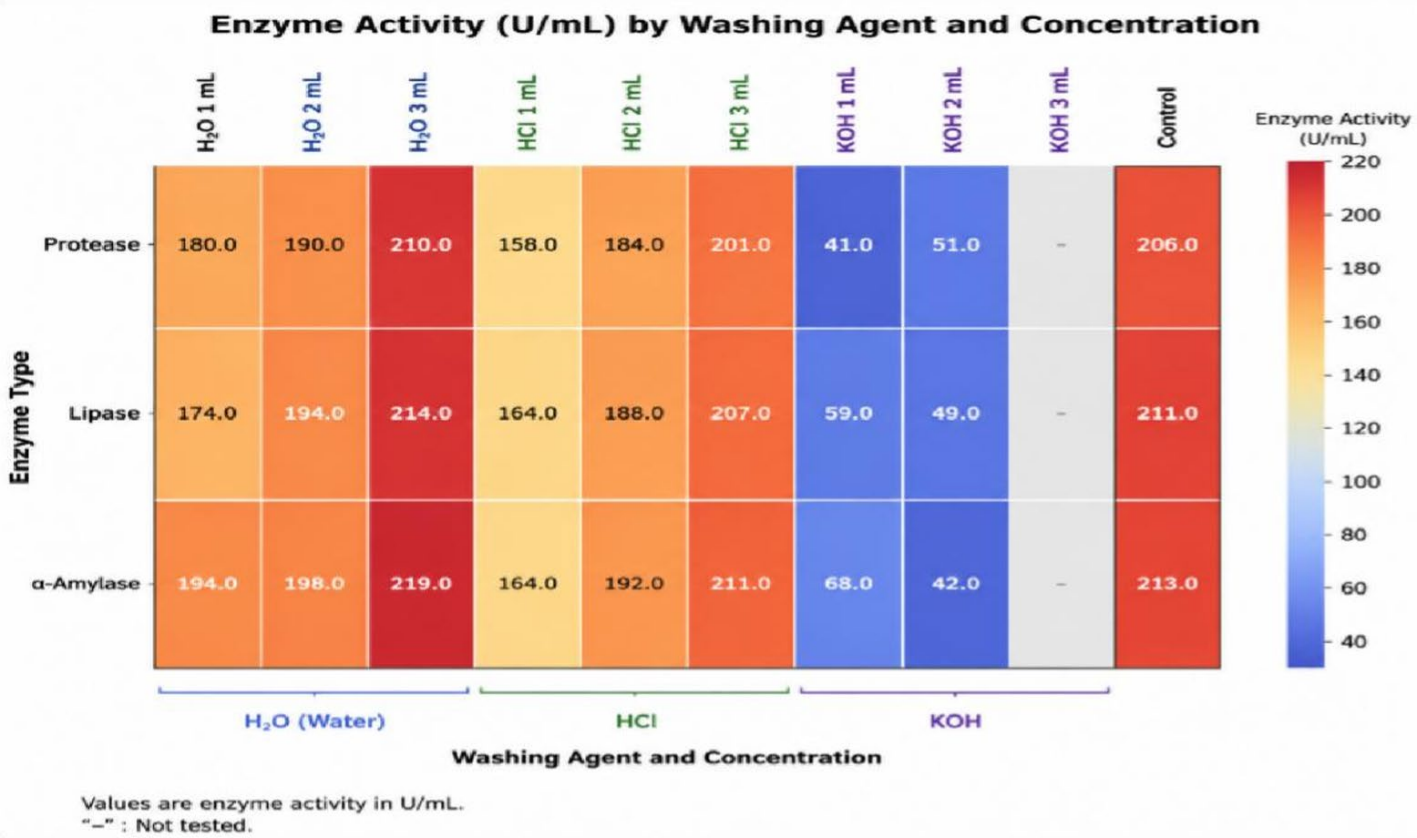
Effect of washing agents on hydrolytic enzyme activities in barley seeds.

In contrast, HCl washing exhibited modest variations in enzyme activities. For instance, protease activity under HCl 3 ml ranged from 201 to 211 U/g FW, which was approximately 6% lower than the control group (206–213 U/g FW), and similar trends were seen in lipase and α-amylase activities. While the enzyme activities under HCl remained relatively stable, they were consistently lower than those observed under water treatments, indicating that acidic conditions did not significantly enhance enzyme functions. KOH washing had the most profound effect on the enzymatic activities, with a noticeable suppression across all enzymes. At KOH 1 ml, protease activity dropped to 41–68 U/g FW, approximately 70–80% lower compared to the control. This decrease intensified with higher concentrations of KOH, reaching non-detectable (ND) levels at 3 ml, demonstrating a complete inhibition of enzyme activity under strongly alkaline conditions. Lipase and α-amylase activities were similarly reduced, with reductions of up to 90% at the highest concentration, further supporting the denaturation effects of alkaline stress. The heat map in Fig. 11 illustrates the impact of different washing agents (H₂O, HCl, and KOH) at 1, 2, and 3 ml at varying concentrations on the activities of protease, lipase, and α-amylase in barley seeds. Water treatment generally enhanced enzyme activity, while both acidic and alkaline treatments, particularly KOH, significantly reduced enzymatic activity at higher concentrations.

These results indicate that while water treatment preserves or slightly enhances enzymatic activity, acidic conditions exert a mild inhibitory effect, and alkaline washing agents cause severe enzyme deactivation. The data suggest that water-based washing appears to be the most suitable for maintaining barley enzyme activities, while acidic and alkaline agents have detrimental impacts, especially at higher concentrations.

## Economic evaluation of regeneration techniques

A practical economic evaluation was performed to compare the three regeneration routes—alkaline (3% KOH), water-based, and acidic (1 M HCl)—for the recovery and reuse of spent activated carbon impregnated with magnesium oxide (MgO). The analysis was conducted for 1 kg of spent activated carbon under Egyptian market conditions, incorporating reagent, energy, and operational costs.In October 2025, and the average market prices in Egypt were approximately as follows:

- Potassium hydroxide (KOH): 120 EGP per kg (≈ 2.45 USD kg⁻¹).
- Concentrated hydrochloric acid (HCl, 32%): 45 EGP per liter (≈ 0.90 USD L⁻¹).
- Electricity: 1.25 EGP per kWh (≈ 0.025 USD kWh⁻¹).
- Distilled water: negligible cost at laboratory scale.

Based on these prices, the estimated total regeneration cost per kilogram of spent AC was calculated as:

- Water-based route: ≈ 25 EGP (≈ 0.50 USD) — lowest cost, minimal reagents, high surface area retention.
- Alkaline route (KOH 3%): ≈ 55 EGP (≈ 1.10 USD) — moderate cost, but provides dual benefits of regenerated AC and nutrient-rich filtrate containing Fe, K, and Mg suitable for use as a liquid fertilizer.
- Acidic route (1 M HCl): ≈ 45 EGP (≈ 0.90 USD) — intermediate cost, effective removal of inorganic residues but reduced pore accessibility.

**Fig. 12:**
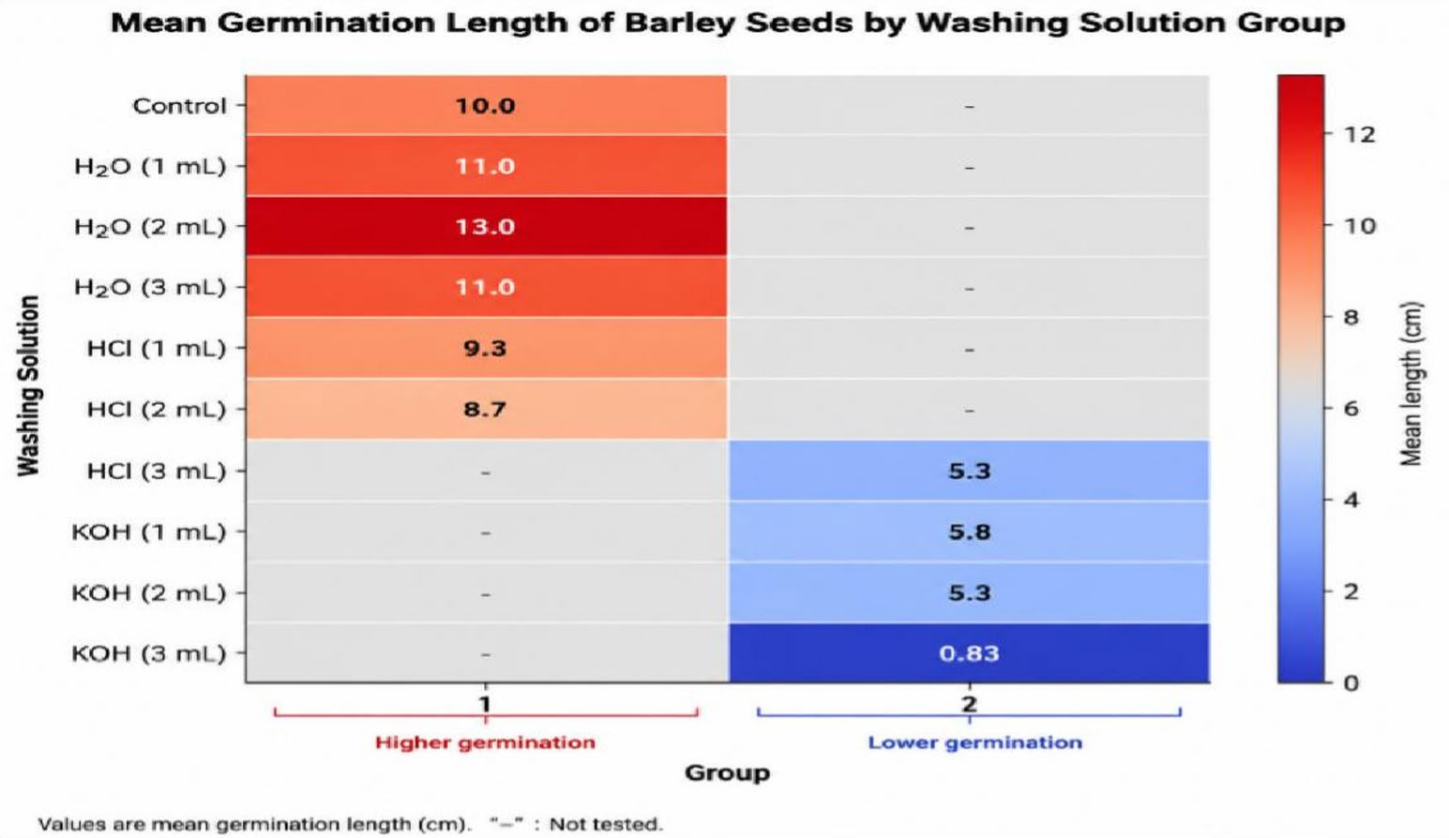
Heat map of the mean germination values for the different washing solutions (H₂O, HCl, and KOH) at different volumes.

For comparison, virgin activated carbon in the Egyptian market costs about 300–400 EGP per kg (≈ 6–8 USD). Therefore, all regeneration methods offer significant economic savings, representing only 10–18% of the cost of new AC. The variance in mean values across various washing solutions and groups is displayed in the heat map (Fig. 12). In Group 1, water treatments produced higher readings, especially at 2 ml (13 cm), while acidic (HCl) and alkaline (KOH) solutions typically produced lower values. The type and concentration of the washing solution had a significant impact, as seen by the lowest mean with KOH at 3 ml (0.83 cm).

The water-based regeneration is the most economical and environmentally friendly option, minimizing chemical consumption while maintaining high textural properties. The KOH-based regeneration slightly increases reagent cost but enhances overall sustainability by generating valuable nutrient-rich effluents that can partially substitute commercial fertilizer use. Hence, under Egyptian economic conditions, both the water- and KOH-based methods are financially and environmentally feasible, supporting circular economy principles through simultaneous waste reduction and material recovery.

## Conclusion

In conclusion, the findings suggest that both water-based and alkaline (KOH) treatments can effectively regenerate spent MgO-impregnated activated carbon derived from exhaust gas purification, each offering distinct advantages for application. Water-based regeneration was the most efficient technique, as it reinstates a substantial surface area and pore structure at a markedly reduced cost compared to virgin activated carbon, rendering it appropriate for extensive applications. Alkaline regeneration enhances the structural properties of carbon and generating nutrient-containing filtrates with potential agricultural relevance after further evaluation within a circular economy framework. Water regeneration represents the most practical and cost-efficient option, restoring a high surface area and pore structure at a cost of less than 10% of virgin AC, indicating its potential suitability for large-scale applications. Acid treatment effectively eliminates inorganic residues; nevertheless, its adverse effects on pore accessibility restrict its applicability for reuse-oriented techniques, making it less suitable for applications that require high surface area and pore structure retention, such as water purification and nutrient recovery. The proposed regeneration methods diminish dependence on virgin activated carbon, reduce waste production, and foster sustainable resource management. The findings demonstrate the potential of incorporating regenerated activated carbon and recovered nutrients into industrial emission control systems and agricultural practices, especially in resource-constrained environments. Such integration supports circular resource management by combining adsorbent reuse with nutrient recovery. The scalability of the proposed regeneration approach depends on optimization of operational parameters, including chemical consumption, energy requirements, and regeneration cycles. At industrial scale, continuous washing and filtration systems could be integrated with existing gas purification units to facilitate adsorbent recovery and reuse. Further pilot-scale studies are required to evaluate long-term performance, process stability, and economic feasibility under real operating conditions. Compared with conventional fertilizer production, the recovery of nutrient-containing washing solutions provides an opportunity to recover valuable elements from waste streams and reduce dependence on external nutrient sources. Compared with commercial activated carbon regeneration approaches, the proposed water-based and alkaline regeneration methods represent lower-cost alternatives while maintaining the structural properties of activated carbon. However, further life-cycle and techno-economic assessments are required for a comprehensive evaluation of environmental and economic performance.

## Supplementary Information

Below is the link to the electronic supplementary material.

Supplementary Material 1

Supplementary Material 2

## Data Availability

Data availability statement: The datasets generated and/or analysed during the current study are available from the corresponding author on reasonable request.
