# Supplementary material for: Valorization of reactivated carbon and recovered nutrients from gas adsorption filters for sustainable fertilization
Source: Sci Rep. 2026 Aug 5;16:24150. doi: 10.1038/s41598-026-64228-x (PMC13442886; doi:10.1038/s41598-026-64228-x)
Supplement: Supplementary file 1 [file 41598_2026_64228_MOESM1_ESM.docx]

**Fig.1.** Steps of Extraction Using KOH and Activation of Activated Carbon: Laboratory Procedure

**Fig.2.** Steps of Extraction Using H_2_O and Activation of Activated Carbon: Laboratory Procedure

**Fig.3.** Steps of Extraction Using HCL and Activation of Activated Carbon: Laboratory Procedur

**Fig.4.** AC samples washed with A: KOH, B: H₂O, and C: HCl were evaluated for porosity using BET

 **Fig.5.** AC samples washed with A: KOH, B: H₂O, and C: HCl were evaluated for isotherms

**Fig.6.** AC samples washed with A: KOH, B: H₂O, and C: HCl were evaluated for BJH

**Fig.7.** AC samples washed with A: KOH, B: H₂O, and C: HCl were evaluated for NLDFT

**Fig.8. Chemical Comparison of AC and solution Phases of Washed Activated Carb**

**Figure 9. Barley seed germination under different washing effluent treatments (a) control, (b) H2O ,(C) KOH and (d) HCL.**

**Fig. 10 Mean germination length (cm) of barley seeds exposed to filtrates produced from washing MgO-loaded activated carbon using different washing solutions.**

**Fig. 11. Effect of Washing Agents on Enzyme Activity in Barley Seeds**

**Fig. 12. The average measurements for the various washing solutions (H₂O, HCl, and KOH) used in the two experimental groups were taken at different volumes.**
