# Supplementary material for: Valorization of reactivated carbon and recovered nutrients from gas adsorption filters for sustainable fertilization
Source: Sci Rep. 2026 Aug 5;16:24150. doi: 10.1038/s41598-026-64228-x (PMC13442886; doi:10.1038/s41598-026-64228-x)
Supplement: Supplementary file 2 [file 41598_2026_64228_MOESM2_ESM.docx]

**Table 1. Sample coding and description of the activated carbon materials used in this study**

| **Sample Code** | **Description** |
| --- | --- |
| Spent AC/MgO | MgO-impregnated activated carbon collected after gas filtration and before regeneration |
| H₂O-AC/MgO | Spent AC/MgO regenerated by water washing |
| KOH-AC/MgO | Spent AC/MgO regenerated by KOH washing |
| HCl-AC/MgO | Spent AC/MgO regenerated by HCl washing |

**Table 2: Textural Properties of AC Samples after Different Washing Treatments (BET Analysis)**

| **Washing Agent** | **BET Surface Area (m²/g)** | **Total Pore Volume (cm³/g)** | **Mean Pore Diameter (nm)** |
| --- | --- | --- | --- |
| **KOH** | **92.45** | **0.1023** | **4.43** |
| **H2O** | **118.18** | **0.1392** | **4.71** |
| **HCL** | **29.49** | **0.0381** | **5.17** |

**Table 3. Comparison of Isotherm Behavior for AC Samples**

| **Washing Agent** | **Isotherm Type** | **Hysteresis Loop** | **Interpretation** |
| --- | --- | --- | --- |
| **KOH** | **Type IV** | **Present (H4-type)** | **Developed micro- and mesoporous structure; improved pore accessibility and adsorption performance** |
| **H2O** | **Type II** | **Absent** | **Limited pore enhancement; weak surface interaction** |
| **HCL** | **Flattened IV** | **Absent** | **Possible pore blockage and reduced pore accessibility; minimal porosity and adsorption performance** |

**Table 4: Textural Properties of AC Samples Based on Washing Method**

| **Washing Agent** | **BJH Surface Area (m²/g)** | **Total Pore Volume (cm³/g)** | **Porosity Type** |
| --- | --- | --- | --- |
| **KOH** | **88.20** | **0.1129** | **Mesoporous** |
| **H2O** | **110.93** | **0.1403** | **Mesoporous** |
| **HCL** | **29.40** | **0.0442** | **Moderate Surface Area** |

**Table 5: Comparison of NLDFT-Derived Pore Structure Parameters of AC Samples**

| **Washing Agent** | **Vp (cm³/g)** | **Peak pore width (nm)** | **Interpretation** |
| --- | --- | --- | --- |
| **KOH** | **0.0875** | **4.3903** | **Developed mesoporous structure** |
| **H2O** | **0.1220** | **4.0864** | **Highest pore volume, mesoporous structure** |
| **HCL** | **0.0131** | **0.7305** | **Strong reduction in pore volume** |

**Table 6: Effect of Washing Agent on Chemical Properties of Solid and Liquid Samples from Activated Carbon**

| **Washing Agent** | **Activated Carbon** | | | | **Solution** | | | **Recommended Use** |
| --- | --- | --- | --- | --- | --- | --- | --- | --- |
|  | **C%** | **N%** | **S%** | **Total Ash (%)** | **Fe %** | **K%** | **Mg%** |  |
| **KOH** | 89.50 | 2.03 | 2.95 | 1.897 | 7.45 | 1.89 | 1.22 | Reuse + Liquid Fertilizer |
| **H2O** | 85.66 | 1.56 | 2.56 | 1.765 | 7.22 | 0.0259 | 1.15 | Balanced Option |
| **HCL** | 80.96 | 1.44 | 2.06 | 1.654 | 6.50 | 0.03 | 1.01 | High Purity (Low Ash) |

**Table 7: Comparison of AC/MgO properties after different washing treatments**

| **Sample** | **BET surface area** | **Total pore volume** | **Pore structure** | **Main observation** |
| --- | --- | --- | --- | --- |
| H₂O washed | Highest among samples | Highest increase | Well-developed micro- and mesoporous structure | Best preservation of pore structure |
| KOH washed | Significant increase | Moderate increase | Developed micro- and mesoporous structure | Enhanced micropore development and nutrient recovery |
| HCl washed | Lowest | Decreased | Limited porosity | Possible pore blockage and reduced pore accessibility |

**Table 8. Comparison of BET and NLDFT Parameters of AC Before and After Regeneration Treatments**

| **Sample** | **BET Surface Area (m²/g)** | **Total Pore Volume (cm³/g)** | **Mean Pore Diameter (nm)** | **NLDFT Pore Volume (cm³/g)** | **Peak Pore Width (nm)** |
| --- | --- | --- | --- | --- | --- |
| Fresh AC | 24.50 | 0.0365 | 3.92 | 0.0271 | 4.3903 |
| KOH | 88.20 | 0.1129 | 3.92 | 0.0875 | 4.3903 |
| H₂O | 110.93 | 0.1403 | 3.92 | 0.1220 | 4.0864 |
| HCl | 29.39 | 0.0442 | 3.92 | 0.0131 | 0.7305 |

**Table 9. Biological grouping of treatments based on germination mean values. Treatments with higher germination means were assigned to Group A, whereas lower-performing treatments were assigned to Group B.**

| **Washing Solution** | **Mean ± SD (cm)** | **Group** |
| --- | --- | --- |
| H2O_2ml | 13.33 | A |
| H2O_1ml | 11 | A |
| H2O_3ml | 10.93 | A |
| Control | 10.33 | A |
| HCL_1ml | 9.33 | A |
| HCL_2ml | 8.67 | A |
| HCL_3ml | 5.33 | B |
| KOH_1ml | 5.83 | B |
| KOH_2ml | 5.33 | B |
| KOH_3ml | 0.83 | B |

**Table 10. Effect of washing agents on hydrolytic enzyme activities in barley seeds (mean ± SD, n = 3)**

| **Treatment** | **Protease (U g⁻¹ FW)** | **Lipase (U g⁻¹ FW)** | **α-Amylase**  **(mg maltose g⁻¹ FW)** |
| --- | --- | --- | --- |
| **Control** | 199.3 ± 12.1 a | 158.0 ± 9.2 a | 0.80 ± 0.07 a |
| **H2O (1 ml)** | 182.7 ± 10.2 ab | 135.0 ± 5.6 b | 0.81 ± 0.04 a |
| **H2O (1ml)** | 194.0 ± 4.0 a | 161.0 ± 3.0 a | 0.88 ± 0.02 a |
| **H2O (3 ml)** | 214.3 ± 4.5 a | 168.0 ± 1.0 a | 0.92 ± 0.04 a |
| **Control** | 199.3 ± 12.1 a | 158.0 ± 9.2 a | 0.80 ± 0.07 a |
| **HCL (1 ml)** | 162.0 ± 3.5 b | 151.0 ± 9.5 a | 0.74 ± 0.06 b |
| **HCL (2 ml)** | 188.0 ± 4.0 ab | 163.3 ± 5.0 a | 0.83 ± 0.06 a |
| **HCL (3 ml)** | 206.3 ± 5.0 a | 168.0 ± 3.5 a | 0.85 ± 0.04 a |
| **Control** | 199.3 ± 12.1 a | 158.0 ± 9.2 a | 0.80 ± 0.07 a |
| **KOH (1 ml)** | 56.0 ± 13.6 c | 53.3 ± 4.0 c | 0.31 ± 0.05 c |
| **KOH (2 ml)** | 47.3 ± 4.7 c | 43.0 ± 7.5 c | 0.24 ± 0.03 c |
| **KOH (3 ml)** | ND | ND | ND |
